# Development of a Wearable Instrumented Vest for Posture Monitoring and System Usability Verification Based on the Technology Acceptance Model

**DOI:** 10.3390/s16122172

**Published:** 2016-12-17

**Authors:** Wen-Yen Lin, Wen-Cheng Chou, Tsai-Hsuan Tsai, Chung-Chih Lin, Ming-Yih Lee

**Affiliations:** 1 Department of Electrical Engineering, Center for Biomedical Engineering, Chang Gung University, Taoyuan 33302, Taiwan; wylin@mail.cgu.edu.tw; 2 Division of Cardiology, Department of Internal Medicine, Chang Gung Memorial Hospital, Taoyuan 33305, Taiwan; ttsai@mail.cgu.edu.tw; 3 Department of Electrical Engineering, Chang Gung University, Taoyuan 33302, Taiwan; sito.19@gmail.com; 4 Department of Industrial Design, Chang Gung University, Taoyuan 33302, Taiwan; 5 Department of Computer Science and Information Engineering, Center for Biomedical Engineering, Chang Gung University, Taoyuan 33302, Taiwan; cclin@mail.cgu.edu.tw; 6 Department of Neurosurgery, Chang Gung Memorial Hospital, Taoyuan 33305, Taiwan; 7 Graduate Institute of Medical Mechatronics, Center for Biomedical Engineering, Chang Gung University, Taoyuan 33302, Taiwan

**Keywords:** wearable, posture monitoring, accelerometer, tilt angle, motion sensing, technology acceptance model (TAM)

## Abstract

Body posture and activity are important indices for assessing health and quality of life, especially for elderly people. Therefore, an easily wearable device or instrumented garment would be valuable for monitoring elderly people’s postures and activities to facilitate healthy aging. In particular, such devices should be accepted by elderly people so that they are willing to wear it all the time. This paper presents the design and development of a novel, textile-based, intelligent wearable vest for real-time posture monitoring and emergency warnings. The vest provides a highly portable and low-cost solution that can be used both indoors and outdoors in order to provide long-term care at home, including health promotion, healthy aging assessments, and health abnormality alerts. The usability of the system was verified using a technology acceptance model-based study of 50 elderly people. The results indicated that although elderly people are anxious about some newly developed wearable technologies, they look forward to wearing this instrumented posture-monitoring vest in the future.

## 1. Introduction

Human posture and activity levels are crucial indices for assessing health and quality of life. Such indices can provide information for targeted health promotion. For example, they can be used to monitor the number of steps walked per day, to remind people to maintain proper postures, and to alert them when they do not maintain healthy postures in sitting, walking, standing positions, and others. Such indices can also be used to assess healthy aging and for the early detection of certain chronic diseases such as Parkinson’s disease [1,2] and stroke [3]. Furthermore, they can be used for health abnormality alerts. The typical applications are fall detection [4,5,6,7] and fall-risk estimation [8], because falling is a major cause of injury and often leads to death in elderly people. The detection of posture change from lying to sitting could even be used to prevent patients or elderly people from bed-fall [9].

Although state-of-the-art sensing systems for posture monitoring are available on the market, such as products from Xsens [10] and APDM [11], some such systems are difficult to set up because they require individual sensor modules strapped on different locations of the body that must be connected to each other (either using wires or wirelessly). Xsens offers an easily configured suit with integrated sensor modules, but it is expensive and nonwashable. The most critical problem of these systems is that they do not offer processing capabilities. All of them must stream sensor data continuously to external units for real-time data processing, and thus they do not offer full mobility. Some features requiring in-system processing and real-time notifications are thus difficult to achieve. Hence, these systems are not suitable for many health care applications, and they especially fail to meet the needs of elderly people.

Studies have focused on integrating various sensing technologies into textile-based clothing. For example, a magnetic induction sensor array was textile-integrated into a shirt, the MAIN Shirt [12], for long-term monitoring of respiration and pulse. In addition, some next-generation pieces of clothing featuring posture and activity monitoring functions have been announced in recent years. However, these types of clothing—such as the Sensatex smart shirt, the Vivonoetics LifeShirt [13], the Fraunhofer FitnessSHIRT, and the E-Health [14] shirt—are designed to monitor multiple vital signs and use only a single simple accelerometer to monitor activities- or sports-related actions. Hence, their posture- and activity-detection capabilities are limited. Accordingly, the development of a highly portable, low-cost solution that could be used both indoors and outdoors to provide health promotion, healthy aging assessments, and health abnormality alerts through posture and activity monitoring is a worthwhile research goal. This goal is expected to become more urgent as medical resources grow increasingly scarce in the future.

This study presents the design and development of a wearable instrumented vest for posture monitoring that was proposed in [15]. This garment can process information, such as the tilting angles of sensors and event detection, internally. The application scenarios and software for this vest are described and developed herein for the purposes of health promotion, healthy aging assessments, and health abnormality alerts. Usability of the vest by elderly people was analyzed through a survey using the technology acceptance model (TAM). Section 2 describes the system design concept and the core technologies developed for this posture monitoring wearable instrumented vest. This section also presents the TAM methodology for usability verification. In Section 3, the fabricated vest is shown, its functional validation test results are presented, and the TAM analysis results are described. In Section 4, some application scenarios are presented for the vest, and the indications from the TAM analysis are discussed. In Section 5, some conclusions and possibilities for future research are discussed.

## 2. Materials and Methods

Section 2.1 reveals the design and development of the wearable instrumented vest for posture monitoring. Section 2.2 describes the usability verification of the vest in the context of the TAM analysis.

### 2.1. Design and Development of the Wearable Instrumented Vest

**Figure 1:**
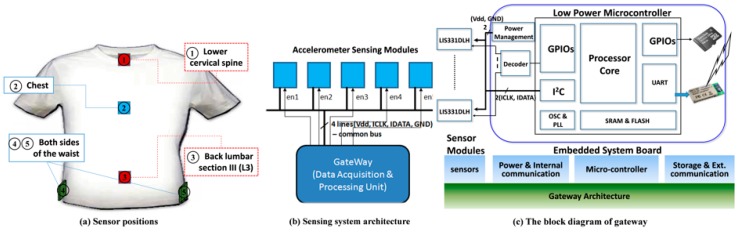
Design of wearable instrumented vest for posture monitoring: (**a**) the sensor positions (dotted lines indicate the sensors at the back of the vest); (**b**) system architecture of the multichannel accelerometer-based sensing system; and (**c**) block diagram of sensing system gateway.

In this study, a wearable instrumented vest for posture monitoring was designed, developed, and fabricated using high-tech textiles at the Taiwan Textile Research Institute (TTRI). Five microelectromechanical system (MEMS)-based accelerometers of model LIS331DLH from STMicroelectronics were deployed on different locations of the garment, as shown in Figure 1a. These three-axis accelerometers were configured with a sensing range of ±2 *g* at a digital data resolution of 12 bits, a sampling rate of 100 Hz, and a sensitivity of 1 mg (1 mg = 2^−10^ g = 1/1024 g, where *g* is the gravitational acceleration). For monitoring multiple postures and activities and for performing gait analysis, these five accelerometers were positioned at the following locations: (1) the lower cervical spine; (2) the middle of the chest; (3) the L3 (lumbar III) section of the back, also known as the center of mass (COM); and (4) the right and (5) left sides of the waist. The lower hem of the vest is elastic and thus the sensors cling to the body, which improves the sensing accuracy.

The sensors positioned at various locations on the vest can be used to detect multiple types of information about the body. For example, the tilting angles of the sensor located on the lower cervical spine provide information on the bending of the neck and upper back. The sensor on the chest detects upper-trunk positions such as sitting, standing, lying, leaning forward while walking, and degree of trunk bending. It can also detect body activities such as sitting up in bed, body turning while sleeping, and trunk bending. The motion sensor at the COM position monitors the static postural balance and ambulatory dynamic stability of the wearer. Gait parameters such as the number of steps, the amount of time spent walking, and the gait symmetry index are measured by detecting motion on both sides of the waist.

At the core of the vest is a multichannel accelerometer-based motion sensing system (Figure 1b). Unlike the systems proposed in most previous studies, which use either a single accelerometer or multiple accelerometers with separate microcontrollers, the current study proposes a system that is capable of controlling multiple accelerometers by using a single control board, a capability that can maintain relatively low levels of cost and power consumption. A single gateway acquires data sequences from the five accelerometers by issuing independent enable signals (en1, en2, en3, en4, and en5) to each accelerometer through a decoder driven by the general-purpose I/O pins (GPIOs) of the microcontroller (ADuC7024, Analog Devices, Inc., Norwood, MA, USA) in the gateway (Figure 1c). The microcontroller not only acquires data from multiple sensors but also performs data processing, information transformation, and event detection. For example, accelerometer information is transformed by a patented low-complexity CORDIC-based tilting angle transformation algorithm [16]. The algorithm is based on CORDIC angle-accumulation (or vectoring mode) operations [17], which were improved for precision enhancement [18]. Therefore, the microcontroller benefits from the use of pure integer iterative add/sub/shift operations in the algorithm and efficiently converts raw data from the three-axis accelerometers to tilting angles [19]. The C program of the proposed CORDIC algorithm, cordic-test.c, can be seen in the supplementary materials. A Bluetooth (BT V2.1 + EDR Class 1) module is connected to the UART interface of the gateway so that the system can communicate wirelessly with external devices such as smart phones or laptop computers, thus rendering it portable.

**Figure 2:**
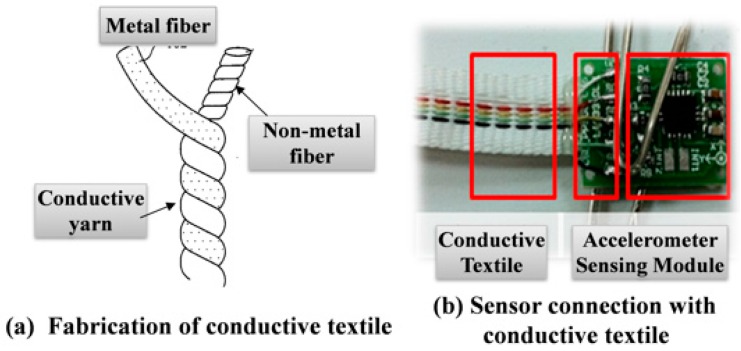
Conductive textile: (**a**) fabrication schematics; and (**b**) integrated with sensing module.

Finally, the aforementioned multichannel motion sensing system and sensor modules are integrated with conductive textiles to fabricate the proposed postural monitoring vest. An elongated metallic fiber is intertwined with a nonmetallic short fiber to form a conductive yarn, which is then woven into a conductive textile (Figure 2a). Figure 2b depicts a photograph of the conductive textile integrated with a bare sensing module board of the proposed vest.

### 2.2. TAM-Based Usability Verification

The TAM, which is one of the most frequently cited models used to explain a user’s perception of a particular form of new technology, has been applied not only to information technology but also to other contexts such as health care technology [20,21,22]. The development of a wearable instrumented vest for posture monitoring could help elderly people realize their posture and activity conditions in a timely manner. However, compared with young adults, elderly people tend to be anxious about using unfamiliar technology. Thus, in the current study, we extended the TAM and added technology anxiety as an external variable to investigate elderly people’s perceptions of the newly designed posture-monitoring vest. Accordingly, we propose the following hypotheses.

- H1: Technology anxiety is negatively correlated with the perceived usefulness of using a posture-monitoring vest.
- H2: Technology anxiety is negatively correlated with the perceived ease of use of a posture-monitoring vest.
- H3: Perceived ease of use is positively correlated with the perceived usefulness of a posture-monitoring vest.
- H4: Perceived ease of use is positively correlated with attitudes toward using a posture-monitoring vest.
- H5: Perceived usefulness is positively correlated with attitudes toward using a posture-monitoring vest.
- H6: Attitude is positively correlated with the behavioral intention to use a posture-monitoring vest.

In total, 50 elderly people residing at the Chang Gung Health and Culture Village were recruited. The inclusion criteria were as follows: (a) age older than 60 years; (b) no structural heart diseases; (c) capable of reading in Mandarin Chinese; and (d) passing the Mini Mental State Examination (MMSE) with a minimum score of 24, which indicates strong mental abilities of memory, attention, and language [23]. Ethical approval was obtained from the Institutional Review Board (IRB) of Chang Gung Memorial Hospital, Taoyuan, Taiwan (104-8175B). Twenty percent of the participants were men. Eighteen percent were 60–69 years old, 36% were 70–79 years old, 42% were 80–89 years old, and 4% were more than 90 years old. Regarding the highest education levels, 14% had graduated from a graduate school, 34% had graduated from a university, 22% had completed high school, 8% had completed junior high school, and 22% had completed elementary school.

A survey was used to confirm the suggested relations of variables relating to the use of wearable technology. The research instrument was a 5-part questionnaire that included users’ perceptions of technology anxiety, ease of use, usefulness, attitude, and behavioral intention regarding wearable technology. The measure for technology anxiety was adapted from Thatcher and Perrewe [24]. Questions regarding perceived ease of use and perceived usefulness were adapted from Davis [25]. Items on attitude and behavioral intention originated from Fishbein and Ajzen [26] and Ajzen [27]. The purpose of this study was explained to the participants before they received the questionnaire. Afterward, the participants answered the MMSE. Only those who achieved an MMSE score of 24 were allowed to participate in the experiment. All participants signed an informed consent form. During the experimental stage, the participants’ demographic data were collected. Subsequently, the participants were required to watch a video describing the functionality of the wearable posture-monitoring vest developed in this study. In the video, the main functions of the vest item (detecting daily activities and postures) were explained. The video presented a simulated scenario exactly representing the vest’s real usage scenario and benefits. To explain how the designed vest could be applied in daily life, after watching the video, we presented the vest to them to improve their understanding of the actual product. Finally, all participants filled in the technology acceptance questionnaire. Each test session took approximately 30 min.

The proposed research hypotheses were evaluated in two stages: measurement model and path analysis. The measurement model, with the use of confirmatory factor analysis, was used to test the reliability and validity of the constructs. Through path analysis, the hypothesized relationships among theoretical constructs were examined. LISREL 8.7 was used for both sets of analysis.

## 3. Results

In this section, the developed wearable instrumented vest for posture monitoring is shown and related testing results are presented. In addition, the results of the TAM analysis of technological acceptance by elderly people are described.

### 3.1. Developed Wearable Instrumented Vest

**Figure 3:**
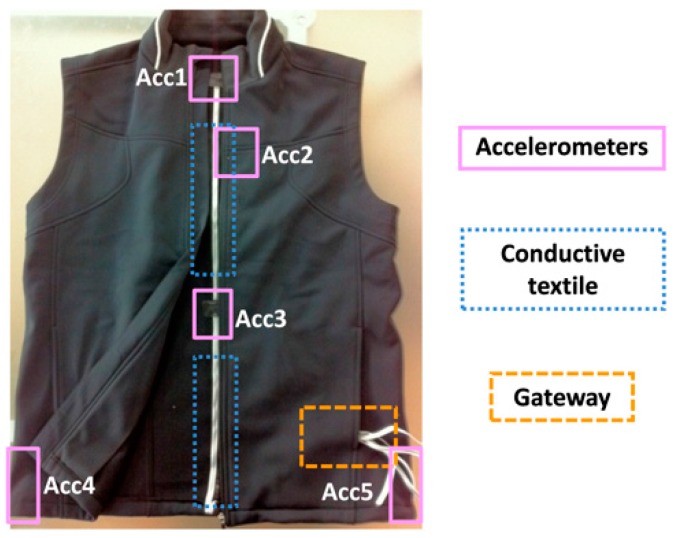
The fully developed wearable instrumented vest.

With the assistance of TTRI, the wearable instrumented vest was fabricated. Five accelerometer modules were embedded on the vest (see Figure 3). They were connected through conductive textiles to the gateway, which could be placed inside a pocket of the vest. The deployment of the system as a vest makes it easy to put on, wear, and take off. To make the vest washable, the embedded sensing modules and gateway were designed for easy removal. System robustness tests and functional validation tests regarding the timing of algorithm execution and accuracy of the tilting angle transformation were conducted carefully.

The robustness of the vest was verified to meet all design specifications, such as conductivity, isolation, wire length of the conductive textile, and motion data sensing and transmission. The test results are summarized in Table 1. To verify that the vest is washable after the removal of all sensing modules and the gateway, the vest was soaked in water with detergent. A brush was applied to remove dirt from the vest, and then the vest was rinsed with clean water and subjected to the spin cycle of a washing machine for initial drying. Finally, the vest was hung out to dry in ambient air for 12 h. Subsequently, all the sensing modules and gateway were plugged back in. All sensors were found to be working correctly. Furthermore, a drifting test was performed to test whether the sensing modules and gateway would continue performing consistently for 12 h. The variation in the sensing data recorded 12 h apart were compared and found to be less than 0.05%.

A previously published validation test [19] for the execution time of the tilting angle transformation algorithm in the gateway was performed to measure the performance and timing efficiency of the algorithm implementation. In addition, the accuracy of the tilting angle transformation was confirmed through testing on a controllable three-axis rotating platform (with a tri-axis step motor controller, TL-3T, TANLIAN E-O Co., LTD, Taoyuan, Taiwan). The maximum error of the tilting angles transformed by the system was within 0.5° of the tilting setting of the rotating platform.

### 3.2. Usability with Technological Acceptance Analysis among Elderly People

To analyze the measurement model, first, the reliability and validity of the questionnaire constructs were assessed according to Cronbach’s alpha values. All constructs had Cronbach’s alpha higher than the recommended value of 0.7, except for the technology anxiety construct, which had a value of 0.664, close to the recommended value. Moreover, the items had item-total correlations ranging from 0.755 to 0.961, which are higher than the threshold value of 0.3. The composite reliability was used to examine the internal consistency. The typical threshold is 0.7. All constructs exceeded this threshold except for technology anxiety, which had a value of 0.6631. Overall, with the exception of technology anxiety, the measurement model of the other constructs was determined to have satisfactory reliability and stable measured results. Subsequently, to validate the measurement model, convergent validity and discriminant validity were determined for the efficiency analysis. For convergent validity to be statistically satisfactory, the composite reliability and average variance extracted (AVE) for each construct should exceed the recommended thresholds of 0.7 and 0.5, respectively. The result of composite reliability has been discussed previously and the score for technology anxiety approached the threshold. The AVE values determined for the perceived usefulness, attitude, and behavior intention constructs were all higher than the threshold value of 0.5, but the values for the technology anxiety (0.4030) and perceived ease of use (0.4559) constructs were not. The discriminant validity was required to meet the criterion that the square root of the AVE of the potentially change items be higher than the correlation coefficients of the other constructs. The square roots of AVE for the potential change items were all greater than the correlation coefficients of the other constructs. This confirmed that the constructs used in this study had favorable discriminant validity. Notably, the results showed that the fit indices for the measurement model were mostly satisfactory.

Path analysis was used to test the six hypothesized relationships among the five variables [28]. As shown in Table 2, H1 states that technology anxiety is negatively correlated with the perceived usefulness of a wearable instrumented vest. The analysis results indicated a path of γ1 = −0.05 (*t* = −0.34, *p* > 0.05), which failed to achieve a level of significance. Thus, H1 was not supported. H2 states that technology anxiety is negatively correlated with the perceived ease of use of a wearable instrumented vest. The analysis results showed a path of γ2 = −0.63 (*t* = −5.65, *p* < 0.001), thus achieving a level of significance of *p* < 0.001. Hence, H2 was supported. H3 states that perceived ease of use is positively correlated with the perceived usefulness of a wearable instrumented vest. The analysis results revealed a path of γ3 = 0.66 (*t* = 4.99, *p* < 0.001), thus achieving a level of significance of *p* < 0.001. Therefore, H3 was supported. H4 states that perceived ease of use is positively correlated with attitudes toward using a wearable instrumented vest. The analysis results indicated a path of γ4 = −0.37 (*t* = 3.25, *p* < 0.01), thus achieving a level of significance of *p* < 0.01. As the result, H4 was supported. H5 states that perceived usefulness is positively correlated with attitudes toward using a wearable instrumented vest. The analysis results revealed a path of γ5 = 0.52 (*t* = 4.59, *p* < 0.001), thus achieving a level of significance of *p* < 0.001. Hence, H5 was supported. Finally, H6 states that attitude is positively correlated with behavioral intention of using a wearable instrumented vest. The analysis results indicated a path of γ6 = 0.81 (*t* = 9.76, *p* < 0.001), thus achieving a level of significance of *p* < 0.001. Therefore, H6 was supported.

## 4. Discussion

### 4.1. Applications for the Wearable Instrumented Vest

**Figure 4:**
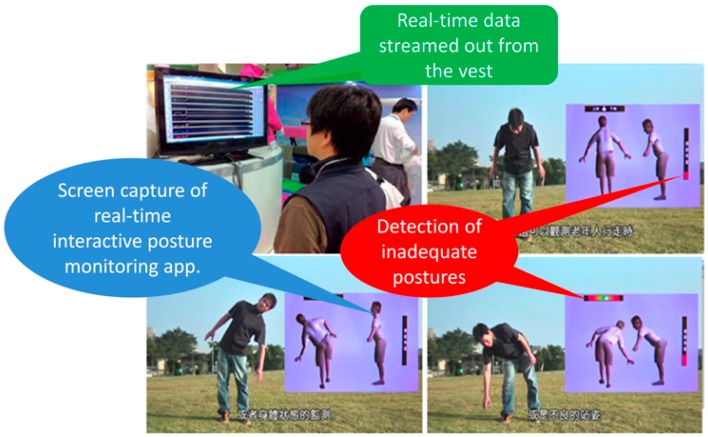
Interactive posture monitoring and real-time warning application.

The developed wearable instrumented vest could be used for various health care applications as introduced in Section 1. Interactive real-time posture-monitoring software was developed for the vest, as shown in Figure 4.

The software receives real-time data (both raw acceleration data and tilting information) from the vest and displays the actual posture of the wearer accordingly. In addition, the application generates real-time warning signals when an abnormal or inadequate posture is detected. For example, when a wearer lowers his or her head while playing a smartphone game for a certain period, or when a wearer with a history of lower back pain bends his or her upper trunk forward by a certain degree and remains in that position for a default amount of time.

**Figure 5:**
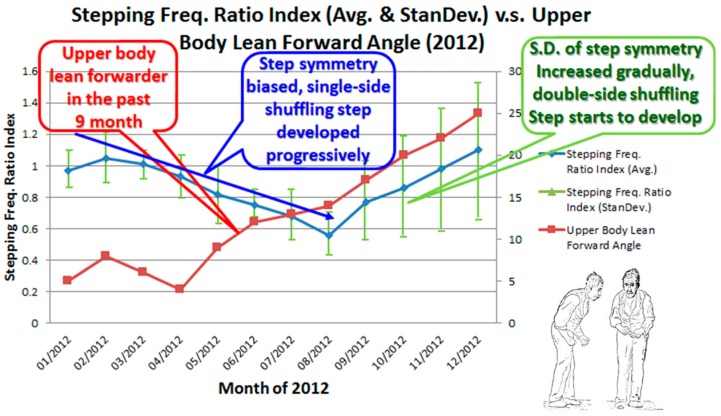
Long-term festination tracking for patients with Parkinson’s disease.

As described in [1], the presented study combined transformed tilting angles of the upper trunk from chest position data with information collected from sensors on both sides of the waist to analyze gait and step symmetry. The Matlab program for the data processing, walktest.m, can be seen in the supplementary materials. These health-dependent parameters can be identified and recorded through the vest by using a developed mobile app. Because the average monthly step symmetry and the angle at which the patient has been leaning forward for the past year can be plotted (see Figure 5), the development of symptoms, such as a festination gait, can be observed and monitored. The blue line indicates the trend of the monthly step symmetry index, the green bar indicates the standard deviation of the step symmetry, and the red line indicates the changes in the average monthly angle at which the upper trunk is inclined forward while walking. The diagram can present a long-term and quantitative tracking record to assist physicians and users seeking to confirm the occurrence of festination gait. The earlier this sign is detected, the earlier the patient can receive preventive medicine as a treatment in the early stages of the disease.

Certain abnormal situations, such as a patient falling to the floor, require immediate alerts. This vest can send such alerts automatically when it detects that a patient has fallen. Moreover, the vest can be used to prevent patients or elderly people from bed-fall as described in [9]. A mobile APP and the detection of lying-to-sitting transitions with the vest were developed to demonstrate this bed-fall prevention scenario.

### 4.2. TAM-Based Usability Analysis for Wearable Instrumented Vest

The TAM was adopted for a survey of elderly people’s perceptions of ease of use, usefulness, technology anxiety, attitude, and behavioral intention regarding the proposed vest. The results showed no significant relationship between technology anxiety and perceived usefulness. Thus, H1 was rejected. However, the remaining hypotheses (i.e., H2–H6) were confirmed. That is, because all participants did not have any experience of using smart clothing in daily life, it can be assumed that the lack of direct experience of wearable technology use might have affected the participants’ perceptions of the vest’s usefulness. The significant path found between technology anxiety and perceived ease of use (PEOU) is consistent with the findings reported in Chang and Im [29] and Guo et al. [30]. The corresponding results revealed that when elderly people have an anxious attitude toward intelligent wearables such as electronic clothing, they consider the proposed wearable instrumented vest to be difficult to use. This TAM survey also explored variables that may affect elderly people’s willingness to use posture-monitoring vests. Perceived ease of use affected perceived usefulness and attitude, and significant paths were determined between perceived usefulness and attitude. These results correspond with the results of previous research studying elderly people’s acceptance of health care technologies [20,30]. The elderly people who considered the technology to be easy to use also perceived the posture-monitoring vest as enhancing their daily lives. Participants who perceived a posture-monitoring vest to be useful and easy to use also gave favorable evaluations of using a posture-monitoring vest. Finally, elderly people who expressed positive attitudes toward the use of a posture-monitoring vest also expressed their willingness to adopt smart clothing. This TAM study showed positive relationships between four variables: perceived ease of use, perceived usefulness, attitude, and behavioral intention.

## 5. Conclusions

This study presents the design and development of a wearable instrumented vest for posture monitoring with multichannel accelerometer-based motion sensing technologies. With the assistance of TTRI, the system was integrated with conductive textile to produce a vest that is suitable for indoor and outdoor wear and has all the same benefits as regular clothing: specifically, it is comfortable, washable, and easy to wear. Validations of all functions of the proposed vest were completed. In addition, integrated software apps were developed and tested to fulfill the proposed targets of health promotion, healthy aging assessments, and health abnormality alerts for healthy aging. Furthermore, a novel garment for cardiac activity monitoring using multichannel seismocardiogram (SCG) technology, as described in [31], for clinical diagnoses of valvular heart diseases and heart failure at home or clinical institutions can be achieved using the same architecture and technologies presented in this study but with different sensor locations and different style of the garment design. A TAM-based survey of 50 elderly people investigated the effect of technology anxiety on their behavioral intention regarding wearable technology. The results verified the usability of the vest and suggested practical implications. Because elderly people often lack experience and knowledge of recent technological advances, they tend to be anxious when facing new technologies. Consequently, it is vital to increase elderly people’s experience with and knowledge of technology to reduce their negative feelings toward using technology. Nevertheless, when asked about their willingness to wear a posture-monitoring vest, most of the participants revealed a positive attitude and even showed willingness to wear such wearable technology in the future.

## Figures and Tables

**Table 1:** Robustness test results: design specification, washability, and signal stability.

Testing Items	Results
**Conductive Textile**	
maximum wire length	90 cm
conductivity	0.2 Ω/10 cm
isolation	Open (>100 MΩ)
**Vest**	
washability (remove all sensing modules and the gateway)	Pass
**Sensors**	
location robustness	Pass
average of $\sqrt{A_{x}^{2}+A_{y}^{2}+A_{z}^{2}}$ in steady state	1 *g* ± 3%
standard deviation of $\sqrt{A_{x}^{2}+A_{y}^{2}+A_{z}^{2}}$	<±3%
drift test (signal variation after 12 h)	<±0.05%

**Table 2:** Summary of hypothesis tests.

Exogenous Variable		Endogenous Variable	Standardized Regression Coefficient	*t*-Value	*p*-Value	Support
Technology Anxiety	→	Perceived Usefulness	−0.05	−0.34	>0.05	No
Technology Anxiety	→	Perceived Ease of Use	−0.63	−5.65	<0.001	Yes
Perceived Ease of Use	→	Perceived Usefulness	0.66	4.99	<0.001	Yes
Perceived Ease of Use	→	Attitude	0.37	3.25	<0.01	Yes
Perceived Usefulness	→	Attitude	0.52	4.59	<0.001	Yes
Attitude	→	Behavioral Intention	0.81	9.76	<0.001	Yes

## Supplementary Materials

The following are available online at http://www.mdpi.com/1424-8220/16/12/2172/s1, C program: cordic-test.c and Matlab program: walkdetect.m.
